# Minimally Invasive Carpal Tunnel Release (CTR) Using the Wongsiri Technique with MiniSURE

**DOI:** 10.1155/2020/6273723

**Published:** 2020-01-04

**Authors:** Sunton Wongsiri, Wongthawat Liawrungrueang

**Affiliations:** Department of Orthopaedic Surgery, Faculty of Medicine, Prince of Songkla University, Hat Yai, Thailand

## Abstract

**Introduction:**

The standard open technique for carpal tunnel surgery has wound problems and complications significantly more than minimally invasive surgery using the Wongsiri technique with MiniSURE Kit® (Surgical Innovation Healthcare Co., Ltd, Bangkok, Thailand) and in particular, the open technique surgery requires a longer time for return to work. CTR surgery with endoscopic devices improves the results with fewer wound problems when compared with the commonly used open technique; however, nerve complications and injury are more prevalent with endoscopic surgery than with the open technique. The Wongsiri technique produces good results with new medical devices such as the MiniSURE View, for improved vision and line-of-sight, and the MiniSURE Cut for improved and complete cutting via the supraretinacular technique that may reduce the nerve problems associated with endoscopic tooling in the carpal tunnel.

**Purpose:**

To evaluate the results of the operation and postoperative outcomes of the Wongsiri technique with a MiniSURE Kit®.

**Methods:**

20 patients underwent carpal tunnel release using the Wongsiri technique and a MiniSURE Kit® with a five-step surgery: MIS starts when the surgeon makes a 1.5–1.8 cm incision, creates a working space, inserts the visual tube of MiniSURE View, inserts the freer, and then cuts the transverse carpal ligament by using the MiniSURE Cut.

**Results:**

All 20 successes of the Wongsiri technique and MiniSURE Kit® surgery occurred within 6.8 minutes operative time and a 12 mm wound size. A single outlier, in one case (6.7%), the patient experienced pillar pain which abated within one month. Patients can return to work in 7.3 days.

**Conclusions:**

The Wongsiri technique with the MiniSURE Kit® demonstrated good outcomes similar to the endoscope. By contrast with the endoscopic surgery, the Wongsiri technique with the MiniSURE Kit® reduced preop, operating, and postop time, many resources, and significant costs and resulted in no nerve problems or complications.

## 1. Introduction

Carpal tunnel syndrome is considered the most common upper limb neuropathy, with a prevalence of 5% in the general population aged 50–60 years with a female/male ratio of 4/1 [1–3]. Carpal tunnel release (CTR) surgery to treat carpal tunnel syndrome is a common operation; five-hundred thousand operations annually occur in the United States of America, with a cost of over two billion U.S. dollars [4]. Standard open carpal tunnel release techniques have been developed with large incisions as much as 3–5 cm, in order to clear the surgical visual field; however, in meta-analysis, it has been shown that 10.2% of standard open CTR operations provide some negative results of wound complications such as painful scars, wound problems, and prolonged return to work [5, 6].

Several minimally invasive (MIS) techniques compete to be the most effective CTR, and surgeons have developed and refined methods to reduce wound complication, painful and prominent scars, and nerve problems [7–9]. Endoscopic carpal tunnel release (ECTR) has shown better results pertaining to wound complication and return to work timeliness when compared to the standard open technique; however, the endoscopic techniques require special training, which includes a long learning curve because of the complicated technique and device, and notably incomplete release incidence may occur [10]. The major noteworthy complications of endoscopic carpal tunnel release are nerve injury, which include transient nerve problem and nerve transection [6, 11]. In contrast, other minimally invasive techniques with limited visualization, with and/or without special tools such as the Indiana Tome (Biomet, Warsaw, USA), the KnifeLight (Stryker Instruments, Kalamazoo, Michigan, USA), and the “Safeguard” system (KMI, Inc., San Diego, USA) have individually shown simplified operative techniques but each also has a limited visual field, and notably, some literature describes safety risks and evidence of incomplete transverse carpal ligament (TCL) release [12–16].

The critical keys of carpal tunnel release are minimally invasive surgery in order to reduce hypersensitivity, soft tissue damage and wound complication, and clear visualization in order to ensure a safe operation and protection of the delicate nerve and vascular structure. The most crucial key of complete TCL release is to prevent a recurrence and an incomplete surgery, so an effective tool is required for complete release.

To enhance the visualization field and to improve efficiency in cutting in MIS, Wongsiri created a new technique (Wongsiri technique) and an innovative set of surgical tools for minimal invasive carpal tunnel release (patent pending), starting with the PSU carpal tunnel retractor in 2008 and culminating in a refinement of the original design with the MiniSURE Kit® in 2013 [8, 17]. The Wongsiri technique with a special designed retractor can improve visualization up to 48.7 mm with a minimal incision [8].

The evolution of a MIS carpal tunnel release tool is the MiniSURE Kit® (Surgical Innovation Healthcare Co., Ltd, Bangkok, Thailand), which is comprised of the MiniSURE View for enhancing visualization and the MiniSURE Cut for a complete and efficient cut of the TCL. The MiniSURE Kit® is a novel, simplified, and combined surgical tool set for carpal tunnel release. The purpose of this study is to describe the preliminary report of the new Wongsiri technique, surgical tips, and early results of minimally invasive carpal tunnel release using a combination of the Wongsiri technique and the MiniSURE Kit®.

## 2. Methods

### 2.1. Study Subjects

This study was approved by Institutional Review Board of Faculty of Medicine, Prince of Songkla University (IRB number EC 56-519-11–1). The twenty patients underwent minimally invasive carpal tunnel release using the novel Wongsiri technique and MiniSURE Kit® at Songklanakarind Hospital, Prince of Songkla University. Nineteen females and one male with a mean age of 55.4 years of age were included in the study. All patients were assessed by specialist hand orthopaedists. The indication of surgery was conservative, failed treatment. Data for the patients collected include demographic data and clinical evaluation as shown in Tables 1 and 2. The operative evaluation comprises operation time, wound sizes, and intraoperative complications as displayed in Table 3. Postoperative results are pain score, wound scar, complications, time of return to work, and satisfaction score as shown in Table 4. Additional phone contact after surgery was utilized in order to retrieve information of clinical and functional conditions and complications.

### 2.2. Surgical Technique

The Wongsiri technique for minimally invasive carpal tunnel release has been consistently performed in Songklanagarind Hospital with the purpose of minimally invasive surgery and to minimize soft tissue injury while simultaneously enhancing the surgical field above the TCL with a special retractor [8, 17].

**Figure 1:**
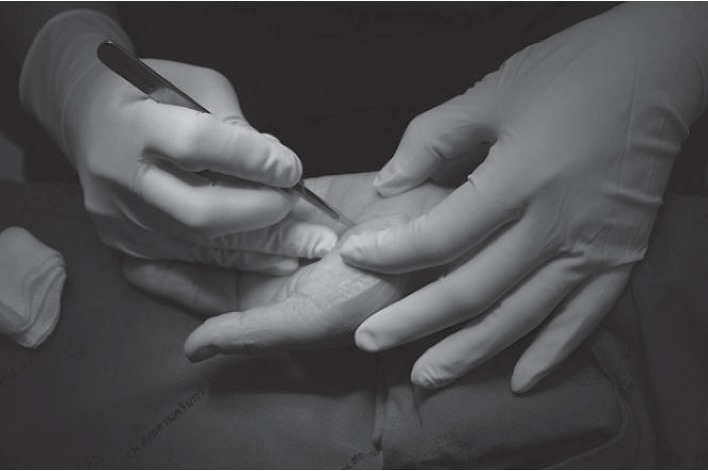
The first step for author's surgical technique.

**Figure 2:**
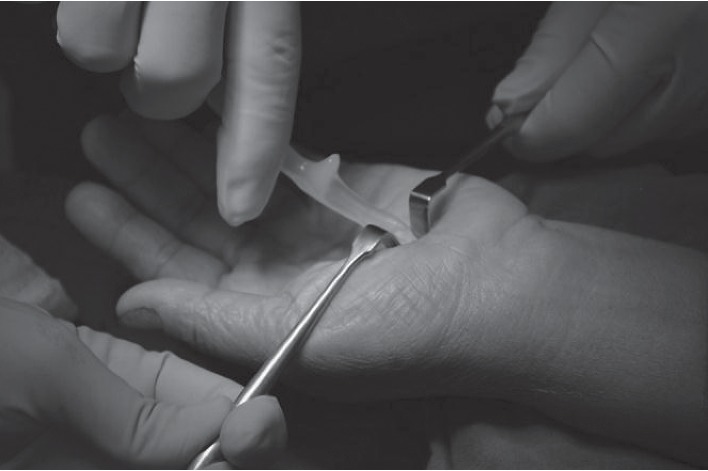
The second step for author's surgical technique.

**Figure 3:**
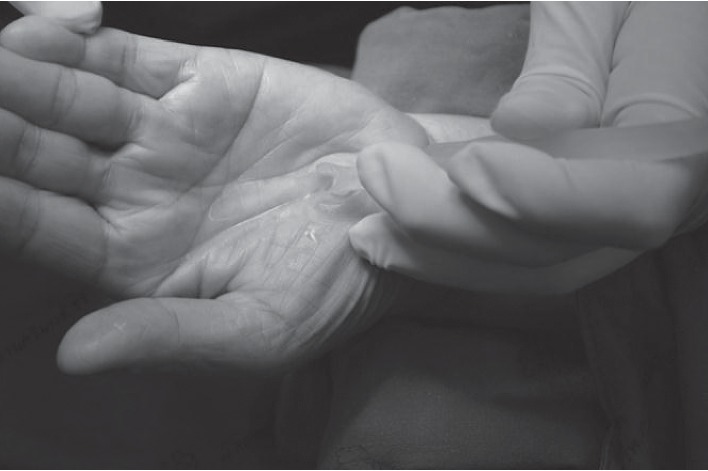
The third step for author's surgical technique.

**Figure 4:**
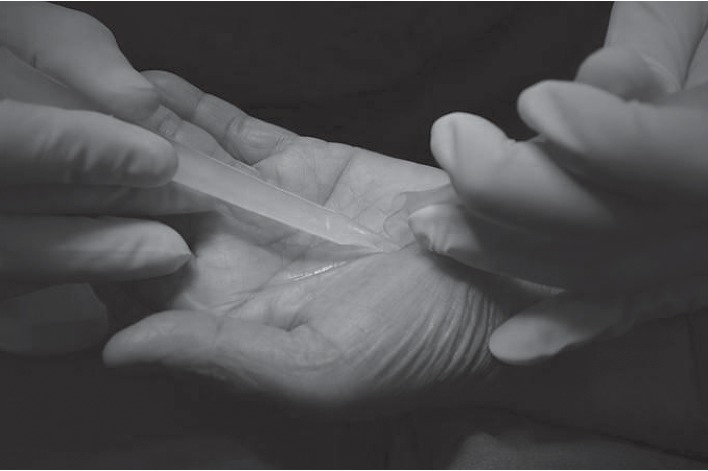
The fourth step for author's surgical technique.

**Figure 5:**
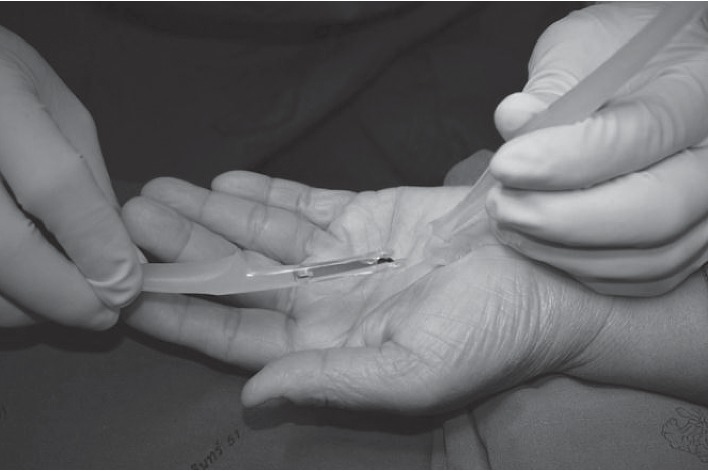
The fifth step for author's surgical technique.

The volar hand is an enriched area with cutaneous branches of the median nerve and ulnar nerve for fine touch sensation. Because of sensitive area of the palmar hand, a large or long incision had been recommended to ensure a safe-zone, and this has led to an interest in a mini-incision and has been introduced in the literature. In order to prudently avoid an injury in this sensitive area, one can utilize a beneficial approach to the area described as beneath the palmaris longus and above retinaculum (BPLAR) as shown below in Figure 1. The insertion of the MiniSURE View above the TCL will crucially enhance the visual field in the BPLAR area. Carpal tunnel decompression performed with a specifically designed knife called the MiniSURE Cut will ensure a complete cut of the TCL. The following comprises the five simplified steps of the Wongsiri technique (available on Youtube at https://youtu.be/7S0M5zVdFgQ) while using the MiniSure Kit®:

- After local anaesthetic, the surgeon makes a 1.5–1.8 cm incision at 2.0 cm distal from the wrist crease in the line of radial axis of the ring finger, through an opening in palmar aponeurosis (Figure 1)
- Surgeon inserts the navigator tip of MiniSURE View to create a working space called the palmaris longus and above retinaculum area that is on top of the TCL (Figure 2)
- Insert the visual tube of MiniSURE View along the created working space so the TCL can clearly be viewed through the visual tube (Figure 3)
- Insert the freer of MiniSURE Cut in order to free the median nerve from adhesion to the TCL (Figure 4)
- Insert the knife of MiniSURE Cut to completely and smoothly cut the TCL (Figure 5)

The MiniSURE Kit® was conceived and developed as a novel tool for minimally invasive carpal tunnel release and is comprised of two useful and practical surgical tools: MiniSURE View and MiniSURE Cut, with four functional components: a navigator tip, a visual tube, an elastic freer, and a surgical knife.

### 2.3. Postoperative Management Protocol

- Over-the-counter medication for pain control and alleviation and an antibiotic for three to five days
- On the third day, the dressing should be changed and the patient should start light hand activities as tolerable
- On the seventh day, the stitches are removed and the patient is encouraged to use hand function as much as possible

## 3. Results

The carpal tunnel operation in this group is female 95%, male 5% with mean age 55.4 years old. Clinical evaluation is shown in Table 2. Most clinical involvement is related to right hand, numbness symptoms, Tinel sign positive, Phalen test positive, and Durkan compression test positive. Operative evaluation is shown in Table 3. Wound scar sizes gradually were reduced in three months. Postoperative evaluation is available for review in Table 4. A minor complication, pillar pain, was found in only one case (6.7%) and became completely cured by the time the patient was re-evaluation after one month. Patients can return to work at 8.7 days by average.

## 4. Discussion

This study shows the success of all twenty carpal tunnel release surgeries which underwent the Wongsiri technique with MiniSURE Kit® and which resulted in less wound complications, less nerve complications, and earlier return to work. In a systematic review [15], the open standard carpal tunnel release (OCTR) showed evidence of more complications and longer return to work than the endoscopic group [6, 18]. Most complications of open carpal tunnel release are wound-related problems such as infection, hypertrophic scarring, or scar tenderness which seem related to wound sizes [19, 20].

Endoscopic carpal tunnel release surgery had shown a significantly earlier recovery than OCTR (8 days for ECTR and 24.6 days for OCTR) and fewer complications (5% for ECTR and 10.2% for OCTR) [6, 19]. However in contrast, 5% of ECTR complications were nerve problems, and the most serious nerve problem was nerve transections [6, 11, 21–23]. In the same manner of minimally invasive surgery, the Wongsiri technique with MiniSURE Kit® is minimally invasive, and it demonstrates a similar result of less wound problems and early return to work as the endoscopic carpal tunnel release technique. Important differences include the working space of the MiniSURE Kit® is out of the carpal tunnel and above the critical TCL, but endoscopic tools are inserted into the carpal tunnel, which seems correlated to higher incidents of nerve problems than other techniques [6, 19, 20, 24, 25]. New endoscopic approaches via the supraretinacular area were introduced to avoid nerve problems by inserting the endoscopic tools above the transverse retinaculum carpal tunnel space from proximal to distal [26]. The author had a similar opinion to avoid nerve problems by working from a perspective outside the carpal tunnel, utilizing the BLAR area approach [8, 17], but assured an incision size, which opened at the distal part, to ensure and provide safety for a critical structure of the recurrence branch of median nerve and superficial palmar arch. Additional benefits should be mentioned, as the Wongsiri technique with MiniSURE Kit® uses little equipment, a small surgical team and no anaesthetic team, minor operation theatre, and hospital disposables and overhead, in-patient rooms and overnight observation are needed, and cost of surgery is reduced.

Other minimally invasive carpal tunnel release tools such as the Indiana Tome (Biomet, Warsaw, USA), the KnifeLight (Stryker Instruments, Kalamazoo, Michigan, USA), and the “Safeguard” system (KMI, Inc., San Diego, USA) have been developed to minimize incision and soft tissue damage [12, 27]. With limited literature, a review of minimally invasive carpal tunnel release with these assistive tools demonstrated an early recovery outcome and minimal complication [12, 28, 29]. However, unlike the Wongsiri MIS technique, those surgeons had a limited visual field during operation and because of this limitation, instances occurred of incomplete TCL release and could not demonstrate effective safety along the compressed area of median nerve [7, 16].

It is the opinion of the author that the Wongsiri technique with the MiniSURE Kit® created value for patients and for health care systems. Patients can easily perceive the better outcome and early back-to-work capability with the benefit of less expensive cost of operation. Health care system expenses will decrease with far fewer human resources, less equipment cost, and shorter time of operation and also patients can access surgery more quickly and receive faster service in an overwhelming health care system. This technique properly addresses effective technology for serving health care systems with minimum invasive benefits.

Limitations of the current study include the small sample of patients and short-term follow-up. In this study, all patients diagnosed had carpal tunnel syndrome without distal radius fractures. However, the reported incidence of median nerve neuropathy associated with distal radius fracture varies greatly in the literature ranging from 0.5 to 21% [30]. The release of the transverse carpal ligament at the time of fracture fixation may reduce the incidence of postoperative median nerve dysfunction [31]. Further studies may apply the MiniSURE Kit® in the case of carpal tunnel syndrome after the traumatic distal end radius fractures.

## 5. Conclusion

The Wongsiri technique using the MiniSURE Kit® for minimally invasive carpal tunnel release has shown success with less complication, less pain, and early time to return to work.

## Figures and Tables

**Table 1:** Demographic data.

Variable	Value
Gender	
Male, number (%)	1 (5)
Female, number (%)	19 (95)
Age	
Years, mean (SD)	55.4 (11.8)

**Table 2:** Clinical evaluation.

Variable	Value
*Affected hand*	
Right, number (%)	8 (40)
Left, number (%)	4 (20)
Both, number (%)	8 (40)
Clinical experience	21.0
Months (SD)	(13.59)

*Clinical symptoms*	
Numbness, number (%)	9 (47.4)
Pain, number (%)	2 (10.5)
Both symptoms, number (%)	8 (42.1)

*Clinical signs*	
Tinel sign positive, number (%)	16 (80)
Phalen test positive, number (%)	16 (80)
Durkan compression test positive, number (%)	17 (85)
Thenar atrophy, number (%)	3 (15)
Sensory deficit, number (%)	2 (10)
FPB weakness, number (%)	2 (10)
APL weakness, number (%)	5 (25)
Opponent weakness, number (%)	2 (10)

**Table 3:** Operative evaluation.

Variable	Value
Operative time, minute (SD)	6.53 (1.09)
Wound size, mm (SD)	12.73 (3.0)
Intraoperative complication, number (%)	0 (0%)

**Table 4:** Postoperative result.

Variable	1-week follow-up	1-month follow-up	3-month follow-up
VAS (SD)	0.25 (0.45)	0.07 (0.26)	0.06 (0.25)
Wound scar, mm (SD)	12 (1.67)	9.73 (2.25)	5.35 (3.96)
Satisfaction, score (SD)	100 (0)	100 (0)	100 (0)

*Complications*			
Total complications, number (%)	0 (0)	1 (5)	0 (0)
Pillar pain, number (%)	0 (0)	1 (5)	0 (0)
Scar tenderness, number (%)	0 (0)	0 (0)	0 (0)
CRPS, number (%)	0 (0)	0 (0)	0 (0)
Wound dehiscence, number (%)	0 (0)	0 (0)	0 (0)

## Data Availability

The data used to support the findings of this study are included within the article.
